# (Benzoato-κ^2^ *O*,*O*′)(quinoline-2-carboxyl­ato-κ^2^ *N*,*O*)(quinoline-2-carboxylic acid-κ^2^ *N*,*O*)manganese(II)

**DOI:** 10.1107/S1600536807066809

**Published:** 2007-12-21

**Authors:** Nuno D. Martins, Joana A. Silva, Manuela Ramos Silva, Ana Matos Beja, Abilio J. F.N. Sobral

**Affiliations:** a CEMDRX, Physics Department, University of Coimbra, P-3004-516 Coimbra, Portugal; b Chemistry Department, University of Coimbra, P-3004-516 Coimbra, Portugal

## Abstract

The crystal structure of the title compound, [Mn(C_7_H_5_O_2_)(C_10_H_6_NO_2_)(C_10_H_7_NO_2_)], contains manganese(II) ions six-coordinated in a distorted octa­hedral environment. The equatorial plane is occupied by four O atoms, two from the carboxyl­ate group of the benzoate ion, the other two from carboxyl­ate/carboxyl groups of the quinaldate/quinaldic acid mol­ecules. The axial positions are occupied by the N atoms of the quinoline ring systems. The metal ion lies on a twofold rotation axis that bisects the benzoate ligand; the quinaldate and quinaldic acid ligands are therefore equivalent by symmetry, and the carboxylate/carboxyl groups are disordered. The complexes are joined together by hydrogen bonds between the carboxyl­ate/carboxyl groups of adjacent quinaldate/quinaldic acid mol­ecules, forming zigzag chains that run along the *c* axis.

## Related literature

For related literature, see Zurowska *et al.* (2007 ▶); Dobrzynska *et al.* (2005 ▶); Kumar & Gandotra (1980 ▶); Catterick *et al.* (1974 ▶).
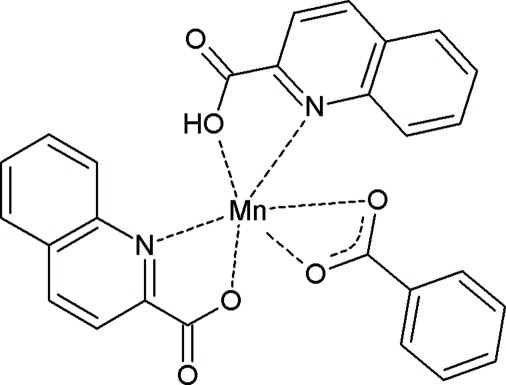


## Experimental

### 

#### Crystal data

[Mn(C_7_H_5_O_2_)(C_10_H_6_NO_2_)(C_10_H_7_NO_2_)]
*M* *_r_* = 521.37
Monoclinic, 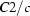

*a* = 19.3839 (4) Å
*b* = 11.6775 (2) Å
*c* = 11.6306 (2) Å
β = 117.288 (1)°
*V* = 2339.67 (8) Å^3^
*Z* = 4
Mo *K*α radiation
μ = 0.61 mm^−1^
*T* = 293 (2) K
0.24 × 0.22 × 0.15 mm

#### Data collection

Bruker APEX CCD area-detector diffractometer
Absorption correction: multi-scan (*SADABS*; Sheldrick, 2000 ▶) *T*_min_ = 0.883, *T*_max_ = 0.908
25798 measured reflections
2917 independent reflections
2413 reflections with *I* > 2σ(*I*)
*R*_int_ = 0.027

#### Refinement

*R*[*F* ^2^ > 2σ(*F* ^2^)] = 0.048
*wR*(*F* ^2^) = 0.149
*S* = 1.08
2917 reflections
169 parameters
1 restraint
H atoms treated by a mixture of independent and constrained refinement
Δρ_max_ = 0.68 e Å^−3^
Δρ_min_ = −0.50 e Å^−3^

### 

Data collection: *SMART* (Bruker, 2003 ▶); cell refinement: *SAINT* (Bruker, 2003 ▶); data reduction: *SAINT*; program(s) used to solve structure: *SHELXS97* (Sheldrick, 1997 ▶); program(s) used to refine structure: *SHELXL97* (Sheldrick, 1997 ▶); molecular graphics: *ORTEPII* (Johnson, 1976 ▶); software used to prepare material for publication: *SHELXL97*.

## Supplementary Material

Crystal structure: contains datablocks global, I. DOI: 10.1107/S1600536807066809/bt2660sup1.cif

Structure factors: contains datablocks I. DOI: 10.1107/S1600536807066809/bt2660Isup2.hkl

Additional supplementary materials:  crystallographic information; 3D view; checkCIF report

## Figures and Tables

**Table 1:** Hydrogen-bond geometry (Å, °)

*D*—H⋯*A*	*D*—H	H⋯*A*	*D*⋯*A*	*D*—H⋯*A*
O2—H2⋯O3^i^	0.96 (7)	1.70 (7)	2.621 (4)	160 (6)

Symmetry code: (i) 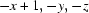.

**Fig. 1.**
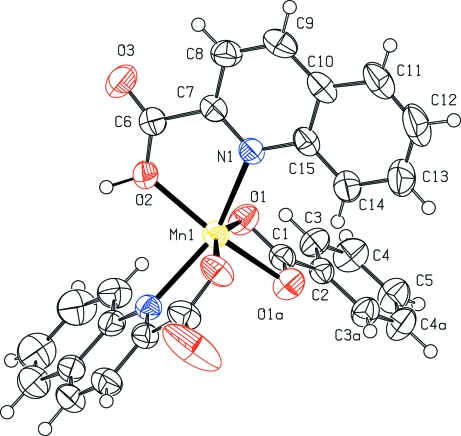
ORTEPII (Johnson, 1976) plot of the title compound. Displacement ellipsoids are drawn at the 50% level.

**Fig. 2.**
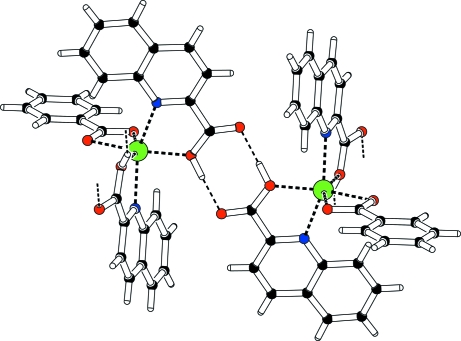
Two of the complexes joined by a hydrogen bond (depicted as a dashed line) between the carboxylic/carboxylate groups of the quinoline molecule.

## supplementary crystallographic information

### Comment

Some compounds with quinoline derivatives, transition metal ions and halide ions exhibit interesting magnetic properties related with the formation of low dimensional elements (Zurowska *et al.*, 2007; Dobrzynska *et al.*, 2005; Kumar & Gandotra, 1980; Catterick *et al.*, 1974). The crystal structure of the title compound, Mn(C_7_H_5_O_2_) (C_10_H_7_NO_2_)(C_10_H_6_NO_2_), consists of manganese(II) ions six-coordinated in a distorted octahedral environment (Fig. 1). The basal plane is occupied by four oxygen atoms with Mn—O distances ranging from 2.1293 (18) to 2.2858 (19) Å. Two basal oxygen atoms belong to the carboxylate group of the benzoate ion, that chelates the metal ion in the usual bidentate mode. Each of the two quinoline molecules supply another O atom to the Mn coordination environment. The apical positions are occupied by the nitrogen atoms of the quinoline ring system, with a distance of 2.2858 (19) Å. Both the benzoic acid and quinoline-2-carboxylic acid molecules are planar. The maximum deviation from the quinolinic plane is 0.1040 (9)Å for O2. The maximum deviation from the benzoic plane is 0.013 (2)Å for O1. The two planes make an angle of 82.98 (9)°. The complexes are joined together by hydrogen bonds, between the carboxylate/carboxylic groups of the quinaldic acid molecules (Fig. 2). The shared hydrogen atom is disordered and the quinoline molecules are statistically neutral or negatively charged. Such H-bonds delineate zigzag chains that run along the *c* axis (Fig. 3).

### Experimental

Approximately 0.13 mmol of 2-quinolinecarboxaldehyde (Sigma, 97%) were dissolved in 2 ml of dimethylformamide and then 0.14 mmol of benzoic acid were added to the solution. 0.12 mmol of manganese chloride tetrahydrated dissolved in 1 ml of water were also added to the former solution. After one month, single crystals of suitable quality were grown from the solution. The refined structure shows that the crystals incorporated a different quinoline derivative than that expected showing that the material purchased from Sigma was contaminated.

### Refinement

All H-atoms were positioned geometrically and refined using a riding model with C—H=0.93 Å, *U*_iso_(H)=1.2*U*_eq_(C). Exception made to the carboxylic hydrogen atom that was first located in a difference map and then refined with a fixed distance to the parent O atom (0.96 Å). This atom is disordered and its occupancy refined to near 0.5 in the first cycles of refinement and it was then fixed to 0.5 in the last cycles.

### Crystal data

**Table:**

[Mn(C_7_H_5_O_2_)(C_10_H_6_NO_2_)(C_10_H_7_NO_2_)]	*F*_000_ = 1068
*M**_r_* = 521.37	*D*_x_ = 1.480 Mg m^−^^3^
Monoclinic, *C*2/*c*	Mo *K*α radiation λ = 0.71073 Å
*a* = 19.3839 (4) Å	Cell parameters from 8901 reflections
*b* = 11.6775 (2) Å	θ = 2.4–27.5º
*c* = 11.6306 (2) Å	µ = 0.61 mm^−^^1^
β = 117.2880 (10)º	*T* = 293 (2) K
*V* = 2339.67 (8) Å^3^	Prism, pink
*Z* = 4	0.24 × 0.22 × 0.15 mm

### Data collection

**Table:**

Bruker APEX CCD area-detector diffractometer	2917 independent reflections
Radiation source: fine-focus sealed tube	2413 reflections with *I* > 2σ(*I*)
Monochromator: graphite	*R*_int_ = 0.027
*T* = 293(2) K	θ_max_ = 28.4º
φ and ω scans	θ_min_ = 2.1º
Absorption correction: multi-scan(SADABS; Sheldrick, 2000)	*h* = −25→25
*T*_min_ = 0.883, *T*_max_ = 0.908	*k* = −15→15
25798 measured reflections	*l* = −15→15

### Refinement

**Table:**

Refinement on *F*^2^	Secondary atom site location: difference Fourier map
Least-squares matrix: full	Hydrogen site location: inferred from neighbouring sites
*R*[*F*^2^ > 2σ(*F*^2^)] = 0.048	H atoms treated by a mixture of independent and constrained refinement
*wR*(*F*^2^) = 0.149	*w* = 1/[σ^2^(*F*_o_^2^) + (0.0806*P*)^2^ + 2.8119*P*] where *P* = (*F*_o_^2^ + 2*F*_c_^2^)/3
*S* = 1.08	(Δ/σ)_max_ < 0.001
2917 reflections	Δρ_max_ = 0.68 e Å^−^^3^
169 parameters	Δρ_min_ = −0.50 e Å^−^^3^
1 restraint	Extinction correction: none
Primary atom site location: structure-invariant direct methods	

### Special details

**Table:**

Geometry. All e.s.d.'s (except the e.s.d. in the dihedral angle between two l.s. planes) are estimated using the full covariance matrix. The cell e.s.d.'s are taken into account individually in the estimation of e.s.d.'s in distances, angles and torsion angles; correlations between e.s.d.'s in cell parameters are only used when they are defined by crystal symmetry. An approximate (isotropic) treatment of cell e.s.d.'s is used for estimating e.s.d.'s involving l.s. planes.
Refinement. Refinement of *F*^2^ against ALL reflections. The weighted *R*-factor *wR* and goodness of fit *S* are based on *F*^2^, conventional *R*-factors *R* are based on *F*, with *F* set to zero for negative *F*^2^. The threshold expression of *F*^2^ > σ(*F*^2^) is used only for calculating *R*-factors(gt) *etc*. and is not relevant to the choice of reflections for refinement. *R*-factors based on *F*^2^ are statistically about twice as large as those based on *F*, and *R*- factors based on ALL data will be even larger.

### Fractional atomic coordinates and isotropic or equivalent isotropic displacement parameters (Å^2^)

**Table:**

	*x*	*y*	*z*	*U*_iso_*/*U*_eq_	Occ. (<1)
Mn1	0.5000	0.20556 (4)	0.2500	0.03799 (18)	
O1	0.48852 (12)	0.37094 (15)	0.14858 (17)	0.0505 (4)	
O2	0.48495 (10)	0.09330 (17)	0.09597 (18)	0.0508 (5)	
H2	0.525 (3)	0.041 (5)	0.103 (7)	0.07 (2)*	0.50
N1	0.37188 (11)	0.15467 (17)	0.15100 (18)	0.0378 (4)	
C1	0.5000	0.4239 (3)	0.2500	0.0395 (7)	
C2	0.5000	0.5526 (3)	0.2500	0.0359 (6)	
C3	0.48852 (16)	0.6123 (2)	0.1396 (2)	0.0457 (6)	
H3	0.4807	0.5728	0.0652	0.055*	
C4	0.4888 (2)	0.7302 (3)	0.1403 (3)	0.0569 (7)	
H4	0.4813	0.7702	0.0664	0.068*	
C5	0.5000	0.7889 (3)	0.2500	0.0582 (10)	
H5	0.5000	0.8685	0.2500	0.070*	
C6	0.41618 (15)	0.0511 (3)	0.0173 (3)	0.0521 (6)	
O3	0.39792 (16)	0.0125 (4)	−0.0885 (3)	0.1252 (15)	
C7	0.35213 (13)	0.0869 (2)	0.0500 (2)	0.0402 (5)	
C15	0.31580 (14)	0.1843 (2)	0.1866 (2)	0.0418 (5)	
C10	0.23875 (14)	0.1435 (2)	0.1169 (3)	0.0487 (6)	
C9	0.22055 (15)	0.0739 (3)	0.0085 (3)	0.0553 (7)	
H9	0.1701	0.0476	−0.0404	0.066*	
C8	0.27651 (15)	0.0452 (2)	−0.0249 (3)	0.0501 (6)	
H8	0.2652	−0.0014	−0.0963	0.060*	
C11	0.18377 (18)	0.1752 (3)	0.1597 (4)	0.0668 (9)	
H11	0.1327	0.1500	0.1148	0.080*	
C12	0.2053 (2)	0.2419 (4)	0.2655 (4)	0.0790 (11)	
H12	0.1690	0.2608	0.2938	0.095*	
C13	0.2808 (2)	0.2828 (3)	0.3327 (3)	0.0710 (9)	
H13	0.2942	0.3292	0.4049	0.085*	
C14	0.33583 (17)	0.2557 (3)	0.2942 (3)	0.0551 (7)	
H14	0.3860	0.2844	0.3389	0.066*	

### Atomic displacement parameters (Å^2^)

**Table:**

	*U*^11^	*U*^22^	*U*^33^	*U*^12^	*U*^13^	*U*^23^
Mn1	0.0367 (3)	0.0369 (3)	0.0406 (3)	0.000	0.0179 (2)	0.000
O1	0.0663 (12)	0.0394 (9)	0.0450 (9)	−0.0012 (8)	0.0248 (9)	−0.0037 (7)
O2	0.0375 (9)	0.0580 (11)	0.0577 (10)	−0.0023 (8)	0.0226 (8)	−0.0194 (9)
N1	0.0344 (9)	0.0405 (10)	0.0380 (9)	0.0031 (8)	0.0162 (8)	0.0023 (8)
C1	0.0338 (15)	0.0405 (16)	0.0427 (16)	0.000	0.0161 (13)	0.000
C2	0.0321 (14)	0.0367 (16)	0.0402 (15)	0.000	0.0178 (12)	0.000
C3	0.0552 (14)	0.0469 (13)	0.0379 (11)	−0.0038 (11)	0.0238 (11)	−0.0019 (10)
C4	0.076 (2)	0.0496 (15)	0.0481 (14)	−0.0032 (14)	0.0305 (14)	0.0087 (12)
C5	0.075 (3)	0.0352 (18)	0.065 (2)	0.000	0.033 (2)	0.000
C6	0.0408 (13)	0.0669 (17)	0.0482 (13)	0.0026 (12)	0.0201 (11)	−0.0100 (13)
O3	0.0565 (15)	0.199 (4)	0.117 (2)	−0.0191 (18)	0.0365 (15)	−0.111 (3)
C7	0.0346 (11)	0.0402 (12)	0.0432 (12)	0.0024 (9)	0.0157 (9)	0.0034 (10)
C15	0.0360 (11)	0.0464 (13)	0.0440 (12)	0.0093 (9)	0.0193 (10)	0.0103 (10)
C10	0.0340 (12)	0.0548 (15)	0.0575 (15)	0.0096 (11)	0.0211 (11)	0.0161 (12)
C9	0.0345 (12)	0.0608 (16)	0.0625 (16)	−0.0049 (11)	0.0152 (11)	0.0050 (14)
C8	0.0415 (13)	0.0508 (14)	0.0497 (14)	−0.0044 (11)	0.0137 (11)	−0.0047 (12)
C11	0.0428 (15)	0.088 (2)	0.078 (2)	0.0158 (15)	0.0348 (15)	0.0171 (18)
C12	0.064 (2)	0.105 (3)	0.088 (2)	0.033 (2)	0.0518 (19)	0.018 (2)
C13	0.069 (2)	0.089 (3)	0.0659 (19)	0.0200 (18)	0.0405 (17)	−0.0030 (17)
C14	0.0501 (15)	0.0647 (17)	0.0522 (15)	0.0115 (13)	0.0249 (13)	−0.0014 (13)

### Geometric parameters (Å, °)

**Table:**

Mn1—O2^i^	2.1293 (18)	C5—C4^i^	1.375 (3)
Mn1—O2	2.1293 (18)	C5—H5	0.9300
Mn1—O1^i^	2.2203 (19)	C6—O3	1.200 (4)
Mn1—O1	2.2203 (19)	C6—C7	1.515 (3)
Mn1—N1	2.2858 (19)	C7—C8	1.405 (3)
Mn1—N1^i^	2.2858 (19)	C15—C14	1.401 (4)
Mn1—C1	2.550 (3)	C15—C10	1.416 (4)
O1—C1	1.258 (2)	C10—C9	1.403 (4)
O2—C6	1.319 (3)	C10—C11	1.416 (4)
O2—H2	0.959 (10)	C9—C8	1.352 (4)
N1—C7	1.319 (3)	C9—H9	0.9300
N1—C15	1.373 (3)	C8—H8	0.9300
C1—O1^i^	1.258 (2)	C11—C12	1.350 (6)
C1—C2	1.503 (5)	C11—H11	0.9300
C2—C3	1.386 (3)	C12—C13	1.390 (6)
C2—C3^i^	1.386 (3)	C12—H12	0.9300
C3—C4	1.377 (4)	C13—C14	1.370 (4)
C3—H3	0.9300	C13—H13	0.9300
C4—C5	1.375 (3)	C14—H14	0.9300
C4—H4	0.9300		
			
O2^i^—Mn1—O2	104.00 (12)	C4—C3—H3	120.0
O2^i^—Mn1—O1^i^	98.44 (7)	C2—C3—H3	120.0
O2—Mn1—O1^i^	157.55 (8)	C5—C4—C3	120.2 (3)
O2^i^—Mn1—O1	157.55 (8)	C5—C4—H4	119.9
O2—Mn1—O1	98.44 (7)	C3—C4—H4	119.9
O1^i^—Mn1—O1	59.13 (9)	C4—C5—C4^i^	120.2 (4)
O2^i^—Mn1—N1	87.81 (7)	C4—C5—H5	119.9
O2—Mn1—N1	73.62 (7)	C4^i^—C5—H5	119.9
O1^i^—Mn1—N1	108.35 (7)	O3—C6—O2	125.5 (3)
O1—Mn1—N1	97.90 (7)	O3—C6—C7	118.1 (2)
O2^i^—Mn1—N1^i^	73.62 (7)	O2—C6—C7	114.0 (2)
O2—Mn1—N1^i^	87.81 (7)	N1—C7—C8	123.7 (2)
O1^i^—Mn1—N1^i^	97.90 (7)	N1—C7—C6	116.9 (2)
O1—Mn1—N1^i^	108.35 (7)	C8—C7—C6	119.3 (2)
N1—Mn1—N1^i^	149.86 (10)	N1—C15—C14	119.0 (2)
O2^i^—Mn1—C1	128.00 (6)	N1—C15—C10	121.0 (2)
O2—Mn1—C1	128.00 (6)	C14—C15—C10	119.9 (2)
O1^i^—Mn1—C1	29.57 (4)	C9—C10—C15	118.3 (2)
O1—Mn1—C1	29.57 (5)	C9—C10—C11	123.2 (3)
N1—Mn1—C1	105.07 (5)	C15—C10—C11	118.5 (3)
N1^i^—Mn1—C1	105.07 (5)	C8—C9—C10	119.9 (2)
C1—O1—Mn1	89.89 (16)	C8—C9—H9	120.1
C6—O2—Mn1	121.28 (15)	C10—C9—H9	120.1
C6—O2—H2	110 (4)	C9—C8—C7	118.8 (3)
Mn1—O2—H2	122 (4)	C9—C8—H8	120.6
C7—N1—C15	118.2 (2)	C7—C8—H8	120.6
C7—N1—Mn1	114.17 (15)	C12—C11—C10	120.1 (3)
C15—N1—Mn1	127.58 (16)	C12—C11—H11	119.9
O1—C1—O1^i^	121.1 (3)	C10—C11—H11	119.9
O1—C1—C2	119.46 (16)	C11—C12—C13	121.2 (3)
O1^i^—C1—C2	119.46 (16)	C11—C12—H12	119.4
O1—C1—Mn1	60.54 (16)	C13—C12—H12	119.4
O1^i^—C1—Mn1	60.54 (16)	C14—C13—C12	120.8 (3)
C2—C1—Mn1	180.0	C14—C13—H13	119.6
C3—C2—C3^i^	119.6 (3)	C12—C13—H13	119.6
C3—C2—C1	120.20 (16)	C13—C14—C15	119.4 (3)
C3^i^—C2—C1	120.20 (16)	C13—C14—H14	120.3
C4—C3—C2	119.9 (2)	C15—C14—H14	120.3
			
O2^i^—Mn1—O1—C1	3.1 (2)	O1—C1—C2—C3	−0.90 (17)
O2—Mn1—O1—C1	−178.80 (10)	O1^i^—C1—C2—C3	179.10 (17)
O1^i^—Mn1—O1—C1	0.0	Mn1—C1—C2—C3	−108 (100)
N1—Mn1—O1—C1	106.70 (10)	O1—C1—C2—C3^i^	179.10 (17)
N1^i^—Mn1—O1—C1	−88.30 (11)	O1^i^—C1—C2—C3^i^	−0.90 (17)
O2^i^—Mn1—O2—C6	85.4 (2)	Mn1—C1—C2—C3^i^	72 (100)
O1^i^—Mn1—O2—C6	−96.5 (3)	C3^i^—C2—C3—C4	0.2 (2)
O1—Mn1—O2—C6	−93.8 (2)	C1—C2—C3—C4	−179.8 (2)
N1—Mn1—O2—C6	2.0 (2)	C2—C3—C4—C5	−0.3 (4)
N1^i^—Mn1—O2—C6	157.9 (2)	C3—C4—C5—C4^i^	0.2 (2)
C1—Mn1—O2—C6	−94.6 (2)	Mn1—O2—C6—O3	160.0 (3)
O2^i^—Mn1—N1—C7	−107.04 (17)	Mn1—O2—C6—C7	−1.8 (3)
O2—Mn1—N1—C7	−1.76 (16)	C15—N1—C7—C8	1.4 (4)
O1^i^—Mn1—N1—C7	154.79 (16)	Mn1—N1—C7—C8	179.9 (2)
O1—Mn1—N1—C7	94.77 (17)	C15—N1—C7—C6	−177.1 (2)
N1^i^—Mn1—N1—C7	−55.92 (15)	Mn1—N1—C7—C6	1.5 (3)
C1—Mn1—N1—C7	124.07 (15)	O3—C6—C7—N1	−163.2 (3)
O2^i^—Mn1—N1—C15	71.34 (19)	O2—C6—C7—N1	0.1 (4)
O2—Mn1—N1—C15	176.6 (2)	O3—C6—C7—C8	18.3 (5)
O1^i^—Mn1—N1—C15	−26.8 (2)	O2—C6—C7—C8	−178.5 (2)
O1—Mn1—N1—C15	−86.85 (19)	C7—N1—C15—C14	179.6 (2)
N1^i^—Mn1—N1—C15	122.46 (19)	Mn1—N1—C15—C14	1.3 (3)
C1—Mn1—N1—C15	−57.55 (19)	C7—N1—C15—C10	−0.1 (3)
Mn1—O1—C1—O1^i^	0.000 (1)	Mn1—N1—C15—C10	−178.43 (17)
Mn1—O1—C1—C2	180.0	N1—C15—C10—C9	−1.4 (4)
O2^i^—Mn1—C1—O1	−178.49 (12)	C14—C15—C10—C9	178.9 (3)
O2—Mn1—C1—O1	1.51 (12)	N1—C15—C10—C11	178.8 (2)
O1^i^—Mn1—C1—O1	180.0	C14—C15—C10—C11	−0.9 (4)
N1—Mn1—C1—O1	−79.27 (12)	C15—C10—C9—C8	1.7 (4)
N1^i^—Mn1—C1—O1	100.73 (12)	C11—C10—C9—C8	−178.6 (3)
O2^i^—Mn1—C1—O1^i^	1.51 (12)	C10—C9—C8—C7	−0.5 (4)
O2—Mn1—C1—O1^i^	−178.49 (12)	N1—C7—C8—C9	−1.1 (4)
O1—Mn1—C1—O1^i^	180.000 (1)	C6—C7—C8—C9	177.3 (3)
N1—Mn1—C1—O1^i^	100.73 (12)	C9—C10—C11—C12	179.6 (3)
N1^i^—Mn1—C1—O1^i^	−79.27 (12)	C15—C10—C11—C12	−0.7 (5)
O2^i^—Mn1—C1—C2	−72 (100)	C10—C11—C12—C13	1.4 (6)
O2—Mn1—C1—C2	108 (100)	C11—C12—C13—C14	−0.6 (6)
O1^i^—Mn1—C1—C2	−73 (100)	C12—C13—C14—C15	−1.0 (5)
O1—Mn1—C1—C2	107 (100)	N1—C15—C14—C13	−178.0 (3)
N1—Mn1—C1—C2	28 (100)	C10—C15—C14—C13	1.7 (4)
N1^i^—Mn1—C1—C2	−152 (100)		

Symmetry codes: (i) −*x*+1, *y*, −*z*+1/2.

### Hydrogen-bond geometry (Å, °)

**Table:**

*D*—H···*A*	*D*—H	H···*A*	*D*···*A*	*D*—H···*A*
O2—H2···O3^ii^	0.96 (7)	1.70 (7)	2.621 (4)	160 (6)

Symmetry codes: (ii) −*x*+1, −*y*, −*z*.

## References

[bb1] Bruker (2003). *SMART* and *SAINT* Bruker AXS Inc., Madison, Wisconsin, USA.

[bb2] Catterick, J., Hursthouse, M. B., New, D. B. & Thornton, P. (1974). *Chem. Commun.* pp. 843–844.

[bb3] Dobrzynska, D., Jerzykiewicz, L. B., Jezierska, J. & Duczmal, M. (2005). *Cryst. Growth Des.* **5**, 1945–1951.

[bb4] Johnson, C. K. (1976). *ORTEPII* Report ORNL-5138. Oak Ridge National Laboratory, Tennessee, USA.

[bb5] Kumar, N. & Gandotra, A. K. (1980). *Transition Met. Chem.* **5**, 365–367.

[bb6] Sheldrick, G. M. (1997). *SHELXS97* and *SHELXL97* University of Göttingen, Germany.

[bb7] Sheldrick, G. M. (2000). *SADABS* University of Göttingen, Germany.

[bb8] Zurowska, B., Mrozinski, J. & Ciunik, Z. (2007). *Polyhedron*, **26**, 3085–3091.

